# Men’s reproductive health knowledge in Mankweng District, Limpopo Province

**DOI:** 10.4102/curationis.v40i1.1732

**Published:** 2017-10-19

**Authors:** Richard M. Rasesemola, Tendani S. Ramukumba, Majapi Masala-Chokwe, Zerish Z. Nkosi

**Affiliations:** 1 Adelaide Tambo School on Nursing, Tshwane University of Technology, South Africa; 2 Department of Health Studies, University of South Africa, South Africa

## Abstract

**Background:**

Gender roles influence men’s attitudes towards reproductive health, and society might assume that reproductive health issues, fertility and family planning are women’s responsibilities. Moreover, literature shows that men have insufficient knowledge about reproductive health matters and some misconceptions about modern contraceptive practices.

**Objectives:**

The aim of the study was to describe reproductive health knowledge of men in Mankweng District and was conducted in 2015.

**Methods:**

A descriptive research study was conducted by using quantitative approach. Data were collected by means of questionnaires. Cluster random multistage sampling was used to select villages in Mankweng District and convenient sampling was used to identify participants who met the inclusion criteria to participate on the study.

**Results:**

A total of 200 questionnaires were distributed and returned; 197 (98.5%) of the returned questionnaires were eligible for analysis. The findings indicated that the majority of participants, 74% (*n* = 145), considered their partners to be unclean during menstruation, and 84.77% (*n* = 167) of participants did not know how to perform self-testicular examination. More than half of the participants, 55.83% (*n* = 67), were not in favour of vasectomy and had strong opinions about it; some of those who reportedly knew what a vasectomy involves had some misconceptions.

**Conclusion:**

The findings showed that despite participants’ reported knowledge about some reproductive health matters their perceptions were unfavourable towards them. Despite men’s sufficient knowledge about sexually transmitted infections (STIs) and ways to protect themselves against STIs men had limited knowledge about other male and female reproductive health matters.

## Introduction

United Nations’ International Conference on Population and Development held in 1994 in Cairo outlined the need to involve men in reproductive health and emphasised men’s shared responsibilities in reproductive health matters for prevention of sexually transmitted infections (STIs), human immunodeficiency virus (HIV) and high-risk pregnancies (United Nations Population Fund 1995:30). Responsibilities entailed shared and equal reproductive and sexual health behaviour such as the utilisation of family planning methods (United Nations Population Fund 1995:30).

Mullick, Kunene and Wanjiru (2005:126) state that most men in South Africa are not actively involved in the reproductive healthcare of their partners and are not typically involved in family planning. They are also not present during labour and delivery of their babies, which has negative outcomes for men and women because it reduces the chances that men will learn and benefit from these reproductive healthcare services. Some of the potential reasons for this include cultural influences that exclude men from issues related to reproductive health and fertility (Mullick et al. 2005:125) and men’s opinion that contraceptives would undermine their authority as head of the households (Matlala & Mpolokeng 2010:40). Little knowledge about contraception has also been cited as one of the reasons why men do not practise contraception (Matlala & Mpolokeng 2010:40).

Societal expectations about what it means to be a man gives men the power to influence and determine women’s reproductive health choices (UNPFA 1995:30), which may undermine women’s ability to protect themselves from unintended pregnancies and from HIV infections (Matlala & Mpolokeng 2010:40).

A study on reproductive health knowledge, attitudes and practices among Iranian and Afghan men living in Tehran showed that men had low scores for knowledge of reproductive health issues; from a group of 578 men only 3.6% (*n* = 17) could give a definition of family planning. In the above-mentioned study the men received information from their partners not from clinics or from educational programmes (Sadeghipour Roudsari et al. 2006:866).

Men’s poor reproductive health knowledge contributes to decreased contraceptive uptake by women (Kabagenyi et al. 2014) and might consequently lead to ill-timed and high-risk pregnancies and high maternal mortality rates. Therefore, the purpose of this study was to describe men’s reproductive health knowledge in Mankweng District.

## Research methods

A descriptive study was conducted in Mankweng District of Polokwane, in Limpopo Province, South Africa.

Mankweng District is a rural area 30 km east of Polokwane Central Business District in Limpopo Province. The Mankweng District’s Traditional Council (Mankweng Traditional Council) has 38 villages with a population of approximately 80 248 (Statistics South Africa 2013). Northern Sotho is the major and first language for the majority of the population (Statistics South Africa 2013). According to the same statistics, Mankweng District had male:female ratio of 87.8:100 (Statistics South Africa 2013). Capricorn District, within which Mankweng is located, has a low couple year protection rate of 52.5% (Massyn et al. 2015:528).

### Population and sampling

The study population comprised of men over the age of 18 who resided in Mankweng District during the time of data collection in June 2015. Multistage sampling was used until the researcher remained with six villages, when convenience sampling was performed by approaching men who met the criteria to be included in the study.

Multistage random sampling was performed as follows:

- Stage 1. Thirty per cent of the villages were sampled out of the list of all villages in Mankweng Traditional Council (38 villages).
- Stage 2. From the 30% (*n* = 11) sampled villages, random cluster sampling was performed until the cluster sample size was six villages.
- Stage 3. All six villages sampled at this level were visited and convenient census sampling was performed, and only 200 men were willing to participate in the study.

### Data collection tool

The researcher designed a questionnaire after an extensive literature review and the Sunrise Model of Culture Care, diversity and universality with cultural and social dimensions (Leininger 2002). The original questionnaire which contained both open-ended and closed-ended questions was in English and field workers translated the contents to the participants who did not understand English and those who were unable to read and write. Instructional guidelines were provided under each section on the questionnaire to guide the participants.

### Data collection

Two male nurses, conversant with the culture and local customs and fluent in Northern Sotho which is the first language for most of the people living in Mankweng District, were recruited as field workers to assist with data collection. They signed confidentiality agreements to ensure confidentiality and privacy of participants. The two field workers assisted in translating and interpreting information leaflets, consent forms and questionnaires to the research participants. They also distributed questionnaires among the research participants, helped research participants who could not read and write to complete the questionnaires and collected completed questionnaires. Data were collected over a period of 3 days: from 05 to 07 June 2015. The participants were recruited from local taxi ranks, the police station, churches, local government offices, house-to-house visits, school visits and alcohol-selling outlets.

### Data analysis

Data analysis included data cleaning and capturing, and testing for associations. Descriptive statistical analysis was used. Data encoding was performed through the use of Excel, and data management and analysis were performed by using Stata V13 programme. Pearson’s chi-square test was used to test for association between any pairs of categorical variables. The interpretation was performed at α = 0.05 level of significance. Descriptive statistics were presented as frequencies and proportions (as expressed in percentages). Responses of open-ended questions were grouped and arranged into themes and were analysed quantitatively.

### Validity and reliability

Development of questionnaire was based on an extensive literature review and the questionnaire was reviewed by the three study supervisors who are reproductive health specialists and experienced researchers.

Pre-testing of the questionnaire was completed by visiting one village that did not form part of the main study, and the questionnaire was administered to 16 men who met the criteria and the responses were assessed to check if the contents of the questionnaire were not prone to misunderstanding or objectionable to participants (Botma et al. 2010:137). Pre-testing assisted the researcher to rephrase some questions to facilitate comprehension of the instrument’s items.

### Ethical considerations

The Departmental Committee for Research and Innovation and Faculty Committee for Research Ethics of the Tshwane University of Technology approved the study protocol and granted permission to the researcher before carrying it out in the community (Ethical clearance reference number: #FRCE 2015/02/004 (2) (SCI))

Informed consent was obtained from each research participant. Transparency was upheld in terms of the objectives of the research, type of data to be collected, method of data collection and the benefits. Those participants who could not read and write provided their thumbprint after a field worker explained the content of the verbal consent form.

The researcher ensured fair and equal treatment of the participants during the study, and there was no victimisation or loss of benefits of the individuals who withdrew participation from the study.

The researcher ensured confidentiality and anonymity through the protection of participants’ identities. The researcher also ensured that no link between the individual identities of the participants to the research could be made.

## Results

Only 200 eligible men agreed to participate in the study. Two hundred questionnaires were distributed to participants over a period of 3 days in different villages in Mankweng District. Two hundred questionnaires were completed, and after checking for completeness a total of 197 questionnaires were suitable for analysis, which accounted for a return rate of 98.5%.

### Biographical information

**FIGURE 1:**
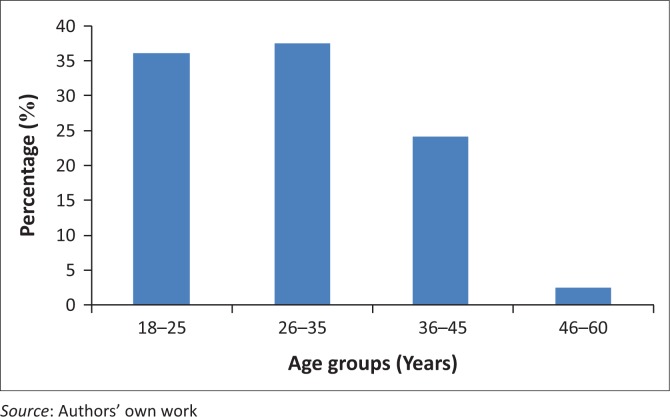
Participants’ age.

Participants’ ages ranged from 18 to 60 years and are shown in Figure 1.

Age group of 26–35 years was represented by 37.50% (*n* = 74.), 18–25 years represented by 36.00 % (*n* = 71), 36–45 years represented by 24.00% (*n* = 47) and 46–60 years was represented by 5 (2.50%) participants.

**FIGURE 2:**
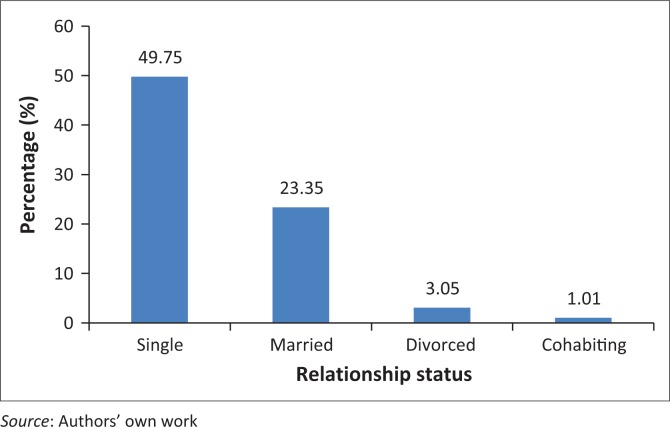
Participants’ relationship status.

Participants’ relationship status is shown in Figure 2.

Almost half, 49.75% (*n* = 98), of the participants were single, 23.35% (*n* = 46) were married, 3.05% (*n* = 2) were divorced and 1.01% (*n* = 1) cohabiting.

**FIGURE 3:**
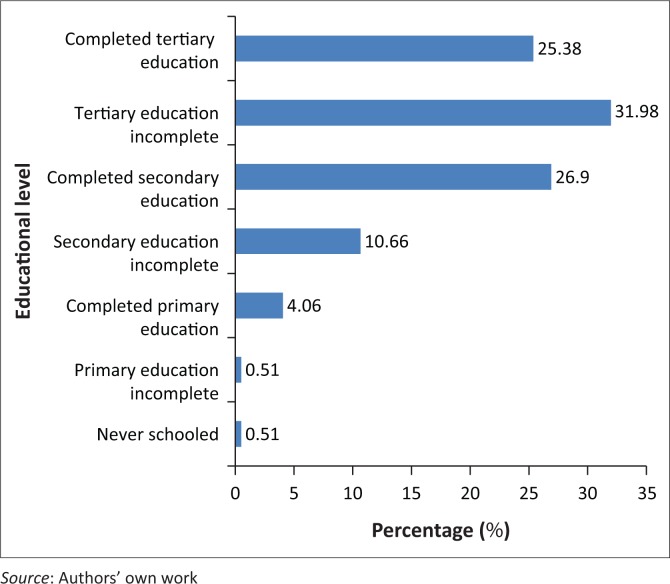
Participants’ educational level.

Participants’ educational levels are shown in Figure 3.

The majority, 84.26% (*n* = 166), of the participants had completed Grade 12 or had obtained educational level beyond Grade 12, with 26.9% (*n* = 53) having completed just Grade 12, 31.98 (*n* = 63) busy with or dropped out of tertiary education and 25.38% (*n* = 50) having obtained tertiary qualification. One participant, 0.51%, had no formal education at all, while another one (0.51%) had not completed primary education.

### Family planning knowledge

Most of the participants, 64.47% (*n* = 127), had no knowledge of what family planning is comprised of, while some, 35.53% (*n* = 70), provided unrelated explanations. Responses related to family planning explanations were quantified and reflected in Table 1.

**TABLE 1:** Family planning-related explanations.

Explanations	*n*	%
Deciding on number of children in the family	20	10.15
Deciding on number of children and use of contraceptives	6	3.04
Use of contraceptives	18	9.14
Discussing and supporting each other	3	1.52
Using condoms to protect oneself	3	1.52
When you don’t have children yet	3	1.52
Planning your family	3	1.52
Child spacing	14	7.11
**Total**	**70**	**35.53**

*Source*: Authors’ own work

Unrelated family planning explanations by the participants include ‘discussing and supporting each other’ at 4.28% (*n* =3), ‘using a condom to protect oneself’ at 4.28% (*n* = 3) and ‘when you don’t have children yet’ at 4.28% (*n* = 3).

While some relevant family planning explanations by the participants included ‘child spacing’ at 20% (*n* = 14) and ‘deciding of a number of children in the family’ at 28.57% (*n* = 20).

### Knowledge of sexually transmitted infections

The majority of participants, 86.29% (*n* = 170), knew the signs of STIs. Although 57.65% (*n* = 98) of those participants had educational levels beyond high school level, there was no significant relationship between participants’ educational levels and their knowledge about STIs (*p =* 0.897).

### Knowledge and opinions about menstruation

Although 34.01% (*n* = 67) of participants thought their partners were unclean during menstruation, 73.60% (*n* = 145) of the participants reported that they knew what is happening to a woman during menstruation. Menstruation-related explanations by the participants who thought their partners become dirty during menstruation are reflected in Table 2.

**TABLE 2:** Menstruation-related explanations.

Explanation	*n*	%
Menstruation allows the body to cleanse itself	2	1.01
Menstrual blood is smelly or unclean	31	15.75
Some churches say so or according to my church, she cannot touch or cook my food during menses	19	9.65
Menstrual blood is dangerous and causes STIs or diseases	2	1.01
Not the right time to have sex during menstruation (‘don’t jump the red robot’)	2	1.01
She does not want to cuddle or bath with me while menstruating	1	0.51
Women also think they are dirty as they bath more than twice when they are ‘on periods’	2	1.01
No explanation	8	4.06
**Total**	**67**	**34.01**

*Source*: Authors’ own work

STIs, sexually transmitted infections.

Among the participants who thought menstruating women are dirty, 15.75% (*n* = 31) reported that menstrual blood is smelly, while 9.69% (*n* = 19) of participants alleged that according to their churches, women may not touch or cook their food during menses. Some participants’ opinions were because of the alleged behaviour of women during menstruation as 0.51% (*n* = 1) participants reported that women do not cuddle with them during menstruation while others reported that women also think they are dirty as they bath more than twice a day during menstruation at 1.01% (*n* = 2).

### Vasectomy related knowledge

Participants were asked about vasectomy and only 60.91% (*n* = 120) of the participants responded to this question. More than half, 55.83% (*n* = 67), of those who responded considered vasectomy not to be good. Vasectomy related motivations by the participants who reported that vasectomy is not good are reflected in Table 3.

**TABLE 3:** Vasectomy related explanations.

Vasectomy related motivations for being ‘not good’	*n*	%
Men will become more promiscuous knowing that they will not impregnate their extramarital partners, and this will lead to increased HIV infection rates	1	0.83
It should be illegal, it is worse than abortion	1	0.83
It will make a man sick	1	0.83
It is castration	1	0.83
No reason	58	48.34
It is dangerous, a man might not get erection anymore	1	0.83
No-go area, a man should bear children and multiply	1	0.83
A man might want to have children later in life	1	0.83
Not necessary because women can continue using contraceptives	2	1.68
**Total**	**67**	**55.83**

*Source*: Authors’ own work

Some participants who reported that vasectomy was not good had lacked understanding about the concept, because of explanations such as: ‘it will make a man sick’ at 0.83% (*n* = 1) and ‘it is dangerous, man might not get erection anymore’ at 0.83% (*n* = 1). Other opinions included: ‘men will become more promiscuous knowing that they will not impregnate their extra-marital partners and this will lead to increased HIV infection rates’ at 0.83% (*n* = 1); ‘vasectomy should be illegal, it is worse than an abortion’ at 0.83% (*n* = 1); and ‘it is castration’ at 0.83% (*n* = 1). Although 44.17% (*n* = 53) of the participants who responded to the question about vasectomy reported that they understood what vasectomy involved, when asked to give reasons some had irrelevant motivation such as ‘it will prevent ejaculation and it is for when a man cannot have erections’.

### Knowledge about circumcision and its association to HIV infections

Almost half of the participants, 49.24% (*n* = 97), knew that circumcision reduces the chances of heterosexually acquired HIV for men while 23.35% (*n* = 46) reported that it does not. The rest of the participants were either unsure or they did not know whether circumcision would reduce their chances of heterosexually acquired HIV or not.

### Testicular self-examination knowledge

The majority of the participants, 84.77% (*n* = 167), did not know how to perform testicular self-examination. There was no significant relationship between the participants’ ages and their knowledge about testicular self-examination (*p =* 0.190), meaning that participants’ ages did not have any influence on testicular self-examination knowledge.

## Discussion

Men usually rely on their partners’ contraceptives use and some might lack knowledge about contraception (Matlala & Mpolokeng 2010:40). In this study, almost two-thirds of the participants had no knowledge of what family planning involved. Some of those participants, who reportedly knew about family planning, provided wrong explanations of family planning. Contraceptive use can substantially improve women’s reproductive health (Peer & Morojele 2013). However, because of men’s misconceptions about contraceptives, women might be prevented from accessing contraceptives. Literature on reproductive health indicated that men complained about contraceptives citing that they lead to premature ejaculation and ageing, loss of libido, body aches and premature death among others (Matlala & Mpolokeng 2010:39). In Nigeria, contraceptive discussions are regarded as women’s affairs by men; however, women are prevented from accessing contraceptive services by their husbands who are the sole decisions makers in matters related to sexuality (Adelekan, Omoregie & Edoni 2014).

Although respondents had little knowledge about family planning, they reportedly had better knowledge about other aspects of reproductive health and STI. In this study, the majority of participants were knowledgeable about the signs of STIs. However, this high level of knowledge about the signs of STIs does not necessarily deter the participants from engaging in unsafe sexual practices. Cuban and Latin American studies discovered that men were knowledgeable about STIs and HIV but continued to ignore methods to protect themselves from contracting STIs and HIV (Rodríguez et al. 2013). The major reason for men to ignore condom use during sexual intercourse is that they say condoms reduce their levels of pleasure (Yourtango 2015). Therefore, they engage in risky unprotected sexual intercourse even though they are knowledgeable that condoms protect them from STIs, HIV and unplanned pregnancies.

Almost a third of the current study’s participants considered their partners to be unclean during menstruation, even though the majority reported to be knowledgeable about menstruation. Garg and Anand (2015) stated that myths and taboos concerning menstruation subject many women to restrictions. In India menstruating girls and women are restricted from offering prayers and touching holy books and they may not prepare food as they are regarded as being unclean and therefore would contaminate the food they prepare. In the West, menstruating women were also considered to be ritually unclean (Wijngaards Institute of Catholic Research 1990). Participants in the current study believed that menstrual blood is dangerous and could cause STIs. While other participants reported that women bathed more often while menstruating, refused to bath and to cuddle with their partners during menstruation. Garg and Anand (2015) further state that the actual cause of menstruation is ovulation followed by a missed chance of fertilisation that leads to bleeding from the endometrial vessels which is followed by preparation for the next cycle; therefore, there is no basis to perceive menstruating women as being unclean or impure.

More than half of the participants who responded to the question about vasectomy were not in favour of vasectomy. Some participants understood what vasectomy entailed; however, they had strong negative opinions about it. Some opinions included the perception that vasectomy promotes promiscuity among males and would lead to increased HIV infection rates based on the opinion that men would not use condom during their extramarital sexual engagements knowing that they would not impregnate their extra-marital partners. While some participants considered vasectomy as worse than abortion and amounting to castration.

Some myths surrounding vasectomy exist and they include: association of vasectomy with de-masculinisation, comparing it with castration, ideas that vasectomy causes painful sex, weight gain and obesity among men and causes men to develop female features, such as breasts, and fears that it would reduce their sex drive and sexual satisfaction (Izugbara & Mutua 2016). Moreover, cultural beliefs in Africa contribute to men’s attitudes towards vasectomies because men’s fertility belongs to the community as a whole, resulting in stigmatising of men who underwent vasectomies (Izugbara & Mutua 2016).

Although there is no scientific evidence to suggest that vasectomy would change a men’s sexual behaviour or attributes, it has not been favoured by men and global contraceptive patterns show that only 2.2% of men in 2013 had this procedure performed (The Conversation 2016).

Male circumcision reduces the risk of heterosexually acquired HIV infection in men by nearly 60% (WHO 2016). Literature on male circumcision and its association with HIV infection showed that the inner skin of the foreskin is more likely to absorb the HIV than any other skin because of its likelihood of sustaining small tears during sex (TUT 2013). Although circumcision reduces chances of contracting HIV for men, it does not provide complete protection against HIV, requiring consistent condom use even after voluntary male medical circumcision (VMMC) (WHO 2016). Less than half of the current study’s participants knew that circumcision reduces the chances of heterosexually acquired HIV for men while almost a quarter of current study’s participants reported that this was not the case. The rest of the participants were either unsure or they did not know about this aspect of VMMC.

Testicular cancer is rare, but it is one of the most common types of cancers in men aged 15–35 years (Testicular Cancer Society 2016). A study to determine knowledge, attitudes and practices of testicular self-examination among male university students from Bangladesh, Madagascar, Singapore, South Africa and Turkey indicated that proportions of participants who performed testicular self-examinations during the past year was highest among South African university students (Peltzer & Pengpid 2015:4741). In the current study, participants were not informed about the importance of routine testicular self-examination as the majority reported that they did not know how to perform this examination.

### Limitations of the study

Some men withdrew their interest for participating in the study after learning that there would not be any incentives for participating. Therefore, because of the poor participation of eligible men in Mankweng, the sample size was realised at 200 men only.

The study was conducted in six villages in Mankweng District among men aged between 18 and 60 years by using a cluster random sampling method. Therefore, the results cannot be generalised to other areas.

Two field workers assisted in the distribution and clarification of the information leaflet, consent form and questionnaire. Inter-rater reliability could not be determined because every respondent completed one questionnaire only. However, the field workers were trained and in cases where they had to interview illiterate participants, they adhered to the questionnaire’s items in the same order.

Participants only completed questionnaires, and those unable to do so were interviewed by two field workers. More in-depth information might have been obtained by conducting individual in-depth interviews.

### Recommendations

‘Education and family-planning programmes in a variety of settings demonstrated that informed individuals everywhere can and will act responsibly in the light of their own needs and those of their families and communities’ (UNPFA 1995:30).

A number of recommendations that arose from the study were as follows:

- There is a need to invest time and resources in educating and empowering men with reproductive health knowledge. This enables men to become equal participants in reproductive health matters, for men to make constructive decisions that will positively impact on couples’ reproductive health, furthermore to meet Sustainable Development Goals three and five which are good health and well-being and gender equality respectively.
- In the light of the need to prevent the rapid spread of HIV and other sexually transmitted diseases and unwanted, ill-timed and high-risk pregnancies, support should be given to existing essential reproductive health education programmes and services for men who emphasise men’s joint responsibilities for couples’ reproductive health matters and help men exercise those responsibilities.
- There is a need to support and strengthen the VMMC campaign and educate men about the importance of VMMC to reduce the risk of contracting heterosexually acquired HIV by men.

## Conclusion

The study described men’s reproductive health knowledge in Mankweng. The findings revealed that despite participants’ reported knowledge about some reproductive health matters their perceptions were unfavourable towards them. Despite men’s sufficient knowledge about STIs and ways to protect themselves against STIs men had limited knowledge about other male and female reproductive health matters. Furthermore, knowledge deficit and misconceptions about reproductive health matters, such as family planning, were identified and described. This may lead to unintended and ill-timed pregnancies and may contribute to increased infant mortality rates. Misconception about vasectomy further associates the procedure with increased HIV rates, and de-masculinisation leading to them not being afforded the authority they deserve in the community. Poor reproductive health knowledge among men delays uptake of contraceptive use by both men and women. Therefore, there is a need to engage men in reproductive health matters in similar manner women have been engaged to ensure that they are informed participants in reproductive matters. Further research needs to be conducted to explore the indigenous reproductive health knowledge in order to develop positive health promotion strategies for men.
